# Neurodevelopmental Disorders Associated with PSD-95 and Its Interaction Partners

**DOI:** 10.3390/ijms23084390

**Published:** 2022-04-15

**Authors:** Amanda M. Levy, Paulino Gomez-Puertas, Zeynep Tümer

**Affiliations:** 1 Kennedy Center, Department of Clinical Genetics, Copenhagen University Hospital, Rigshospitalet, 2600 Glostrup, Denmark; marie.amanda.bust.levy@regionh.dk; 2 Molecular Modeling Group, Severo Ochoa Molecular Biology Centre (CBMSO, CSIC-UAM), 28049 Madrid, Spain; pagomez@cbm.csic.es; 3 Department of Clinical Medicine, Faculty of Health and Medical Sciences, University of Copenhagen, 2200 Copenhagen, Denmark

**Keywords:** postsynaptic density, DLG4, epilepsy, synaptopathy, clinical genetics, excitatory synapses, intellectual disability, glutamate signaling

## Abstract

The postsynaptic density (PSD) is a massive protein complex, critical for synaptic strength and plasticity in excitatory neurons. Here, the scaffolding protein PSD-95 plays a crucial role as it organizes key PSD components essential for synaptic signaling, development, and survival. Recently, variants in *DLG4* encoding PSD-95 were found to cause a neurodevelopmental disorder with a variety of clinical features including intellectual disability, developmental delay, and epilepsy. Genetic variants in several of the interaction partners of PSD-95 are associated with similar phenotypes, suggesting that deficient PSD-95 may affect the interaction partners, explaining the overlapping symptoms. Here, we review the transmembrane interaction partners of PSD-95 and their association with neurodevelopmental disorders. We assess how the structural changes induced by *DLG4* missense variants may disrupt or alter such protein–protein interactions, and we argue that the pathological effect of *DLG4* variants is, at least partly, exerted indirectly through interaction partners of PSD-95. This review presents a direction for functional studies to elucidate the pathogenic mechanism of deficient PSD-95, providing clues for therapeutic strategies.

## 1. Introduction

**Figure 1:**
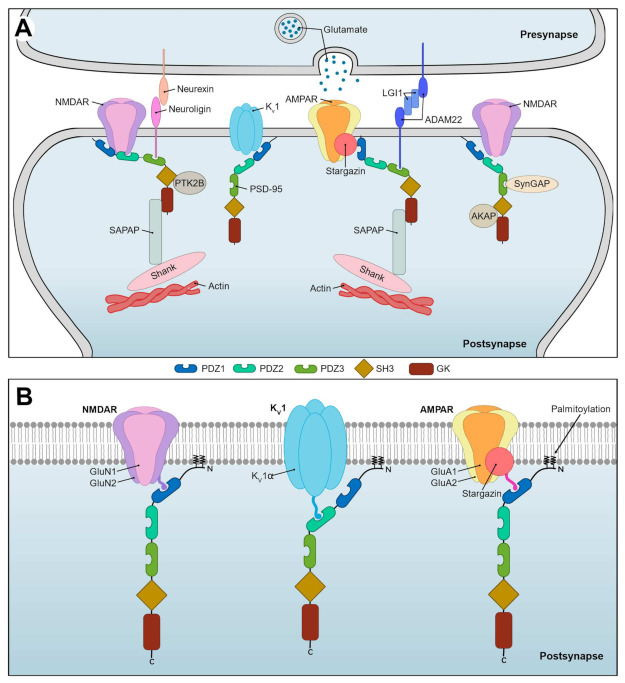
Simplified schematic overview of the major PSD-95 interactions in the postsynaptic density. (**A**) PSD-95 anchors K_v_1 channels, NMDA receptors, and AMPA receptors (through stargazin) to the postsynaptic membrane as they bind the first two PSD-95/Discs large/Zonula occludens 1 (PDZ) domains of PSD-95, whereas neuroligin and ADAM22 bind the third PDZ domain. Amongst others, PSD-95 also binds PTK2B, which is recruited to the NMDAR-PSD-95-complex through binding to PSD-95 SRC homology 3 (SH3) domain, AKAP, which links the NMDAR–PSD-95-complex with signaling enzymes (not shown), and SynGAP, which binds all three PSD-95 PDZ domains. Furthermore, SAPAP binds PSD-95 guanylate kinase-like (GK) domain and actin-binding Shank, forming a scaffolding protein complex, which anchors the actin cytoskeleton to the postsynaptic membrane. Transsynaptic interactions include the neuroligin–neurexin interaction as well as the ADAM22–LGI1–complex. The location of the latter is based on one of the models proposed by Fukata et al. [3]. (**B**) Representative diagram of the structure of NMDAR, AMPAR, and K_v_1 as well as their interaction with PSD-95, which is anchored to the postsynaptic membrane through N-terminal palmitoylation. NMDARs most often comprise two obligate GluN1 subunits (pink) and two GluN2 subunits (purple), although they also exist as triheterotetramers (GluN1–GluN1–GluN2–GluN3; not shown). Various K_v_1α-subunits assemble in homo- or heterotetramers to form the K_v_1 channels. The most common AMPAR subunit composition includes two GluA1 (orange) and two GluA2 subunits (yellow), although they may also include GluA3 and/or GluA4 subunits. N, N-terminal; C, C-terminal.

The human brain consists of billions of neurons, which are highly interconnected through synapses. On the postsynaptic side of excitatory synapses resides the postsynaptic density (PSD), a massive semi-membrane-associated protein complex, dynamic in its morphology and composition, and important for synaptic strength and plasticity [1]. The PSD is highly enriched with scaffolding proteins of the discs large (DLG) subfamily, belonging to the membrane-associated guanylate kinases (MAGUKs). This four-member subfamily comprises SAP97, PSD-93, and SAP102 (encoded by *DLG1*, *DLG2*, and *DLG3*, respectively) as well as PSD-95 (encoded by *DLG4*), which is the prototypic and most abundant MAGUK in the PSD (Table 1) [2]. PSD-95 clusters in postsynaptic nanodomains, where it binds to the ionotropic glutamate receptors AMPARs and NMDARs (α-amino-3-hydroxy-5-methyl-4-isoxazolepropionic acid and *N*-methyl-D-aspartate receptors, respectively), Shaker-type voltage-gated potassium (K_v_1) channels, the cell adhesion molecules neuroligins (NLGNs), and the transmembrane protein ADAM22 (a disintegrin and metalloprotease 22) (Figure 1A, Table 1) [3,4]. Through modeling and super resolution imaging, these nanodomains have been shown to align directly across from presynaptic nanodomains enriched with scaffolding proteins and neurotransmitter release machinery [3,5], leading to the suggestion of functional transsynaptic nanocolumns, primarily organized by two protein complexes: one containing post- and presynaptic NLGN1 and neurexins, respectively; and another comprising the secreted neuronal protein LGI1 (leucine-rich glioma-inactivated protein 1) and ADAM22 (Figure 1A) [3,6,7]. PSD-95 is central in the organization of these transsynaptic nanocolumns as well as PSD architecture, and as the latter is known to control fluctuation and amplitude of postsynaptic currents, PSD-95 is ultimately an essential regulator of the plasticity and strength of excitatory signaling [8,9,10].

*DLG4*-related synaptopathy is a rare brain disorder characterized by intellectual disability (ID), developmental delay (DD), autism spectrum disorder (ASD), attention deficit hyperactivity disorder (ADHD), and epilepsy, associated with pathogenic *DLG4* variants [11]. As PSD-95 primarily functions as a hub for key components of the PSD and the transsynaptic nanocolumns, it is possible that the clinical symptoms of *DLG4-* related synaptopathy are, at least in part, caused by PSD-95-dependent malfunction of its interaction partners. Here, we review some of the main PSD-95 binding partners as well as their association with neurodevelopmental disorders (NDDs).

## 2. PSD-95 and the Synapse

Protein domains of all the members of the DLG subfamily are arranged in a modular fashion and comprise three PSD-95/Discs large/Zonula occludens 1 (PDZ) domains followed by one SRC homology 3 (SH3) and one guanylate kinase-like (GK) domain [2], partitioned into two independent supramodules: one containing the first two PDZ domains (PDZ1-2) and the other, the PDZ3, SH3, and GK domains [12,13]. These supramodules can modulate the ligand-binding affinity through intra- and interdomain dynamics and structural rearrangements [13,14]. The PDZ domains of PSD-95 contain a canonical binding pocket, which its ligands associate with through a PDZ-binding motif (PBM) in their C-termini [15]. In addition, residues outside the binding pocket of PDZ domains modulate ligand-binding, which may explain the protein-specific preference for individual PDZ domains [16].

PSD-95 is orientated perpendicular relative to the synaptic membrane, which enables it to create a link between proteins in the membrane and those embedded in the PSD (Figure 1A) [1]. The latter group includes, amongst others, SynGAP (synaptic Ras-GTPase activating protein), which binds to the PDZ1-3 domains; PTK2B (protein tyrosine kinase 2 beta), which binds to the SH3 domain when recruited to the NMDAR-complex residing at the membrane; and AKAPs (A kinase anchoring proteins), which bind to the SH3 and GK domains creating a link for signaling enzymes targeting NMDARs [17,18,19]. In addition, the GK domain-binding SAPAP (SAP90/PSD-95-associated protein; also known as GKAP) connects the scaffolding protein Shank (SH3 and multiple ankyrin repeat domain) to PSD-95, forming the Shank–SAPAP–PSD-95 scaffolding complex [20,21,22]. As Shank interacts with the actin cytoskeleton, the scaffolding complex anchors the membrane to the cytoskeleton and is thus crucial for PSD architecture (Figure 1A) [2]. This is in line with the observation that knockdown of PSD-95 leads to a fragmentation of the PSD [23]. Similarly, simultaneous knockdown of PSD-95, PSD-93, and SAP102 decreases PSD size and synaptic transmission, and increases the number of silent synapses [24], which is in accordance with the reciprocal compensatory abilities of DLG subfamily members [25].

PSD-95 is the target of many types of posttranslational modifications (PTMs) known to modulate its postsynaptic localization as well as glutamate receptor activity, dendritic spine growth, and synaptic plasticity [26]. Dynamic palmitoylation–depalmitoylation cycles of PSD-95 regulate synaptic retention of AMPAR and NMDAR, as N-palmitoylation leads to clustering of PSD-95 in nanodomains in dendritic spines [27,28,29]. Various pathways and proteins can then in turn modulate the palmitoylation of PSD-95, contributing to the dynamic localization of PSD-95 [30,31,32]. Likewise, phosphorylation of PSD-95 also affects clustering and protein–protein interactions [26,33]. Other types of PTMs affecting PSD-95 function include S-nitrolyzation, ubiquitination, and neddylation [26].

Overall, the architecture of the PSD and the nanodomains as well as the interactome of PSD-95 are all highly complex and various modulations and interactions affect several synaptic pathways, important for functional synapses and signaling.

## 3. PSD-95 Binding Partners and Their Involvement in Neurodevelopmental Disorders

PSD-95 is a pivotal scaffolding protein in the PSD and acts as a hub and anchor for several proteins involved in various functions and pathways. Here, we highlight some of the transmembrane proteins whose interaction with PSD-95 is well established and which are implicated in NDDs and epilepsy.

### 3.1. Shaker-Type Voltage-Gated Potassium Channels

The K_v_1 channel subfamily comprises eight different pore-forming α-subunits (K_v_1α), of which K_v_1.1, K_v_1.2, and K_v_1.4 (encoded by *KCNA1*, *KCNA2*, and *KCNA4*, respectively; Table 1) are the most abundant in the mammalian brain [34,35]. α-Subunits assemble in homo- or heterotetramers and, together with auxiliary K_v_β-subunits, form K_v_1 channels (Figure 1B). These regulate the duration, frequency, and amplitude of action potentials, neurotransmitter release, and resting membrane potential, the efficiency of which is dependent on localization and clustering of the channels [34]. This is primarily mediated through interaction with PSD-95, where the two first PDZ domains recognize the PBM in the C-terminal of all K_v_1α-subunits and anchor the channels to postsynaptic sites (Figure 1) [4,36]. Whereas the interaction between PSD-95 and K_v_1.4 is well established, the relatively weaker binding of PSD-95 to K_v_1.1 and K_v_1.2 is less described [4,37]. This affinity difference is suggested to be due to individual PBM sequences; where the ETDV motif of K_v_1.4 results in a strong binding, the hydrophobic leucine in the LTDV motif of K_v_1.1 and K_v_1.2 is likely to reduce the affinity to PSD-95 [37].

All of the genes encoding the above-mentioned K_v_1α-subunits are associated with NDDs. For *KCNA4*, only a single missense variant segregating with ID, ADHD, and abnormal striatum in a consanguineous family is described [38]. On the other hand, *KCNA1* and *-2* variants are clearly linked to various NDDs and epilepsies. *KCNA1* variants are typically associated with episodic ataxia type 1, and have in rare cases been associated with paroxysmal dyskinesia and epilepsy [39]. Both gain- and loss-of-function (GoF and LoF, respectively) variants in *KCNA2* lead to cerebellar dysfunction, resulting in different neurological and -developmental disorders where the clinical picture is dominated by early-onset developmental and epileptic encephalopathy (DEE), ID, as well as ataxia and other movement disorders [40].

### 3.2. AMPA Receptors

In the mammalian brain, AMPARs are responsible for fast excitatory synaptic signaling and are important for synaptic plasticity [41]. They are concentrated in the PSD, but their presence in intracellular pools and at extrasynaptic sites allows for the regulation of synaptic transmission through recycling and lateral diffusion [42,43]. AMPARs are composed of the pore-forming subunits GluA1–4 (encoded by *GRIA1–4*, respectively; Table 1; Figure 1B), each of which consists of four domains: the N-terminal domain (NTD), the ligand-binding domain (LBD), the transmembrane domain (TMD), and the C-terminal domain (CTD) [41]. The extracellular NTD is involved in subunit assembly, synaptic localization, and receptor clustering [44,45,46]. The LBD undergoes conformational changes induced by ligand-binding and is responsible for channel gating [47,48]. The TMD conducts cations when the channel is open, and the cytoplasmic CTD is involved in receptor anchoring, signaling, and trafficking [41,49].

Although homotetrameric AMPARs have been described, they typically assemble as heterotetramers, and the subunit composition often corresponds to the localization of the receptor (Figure 1B). GluA2 is the predominant subunit followed by GluA1, GluA3, and then GluA4 [50]. The subcellular localization of different AMPAR pools is dynamic, as is evident from the insertion of GluA1/GluA2-containing AMPARs into the postsynaptic membrane upon induction of synaptic plasticity, which are then replaced by GluA2/GluA3-containing AMPARs at rest [51,52]. Traditionally, GluA2-containing AMPARs are considered Ca^2+^-impermeable and mainly conduct K^+^ and Na^+^-ions, whereas AMPARs that lack GluA2 are Ca^2+^-permeable. However, recent evidence challenges this binary classification and suggests that Ca^2+^-permeability is primarily dependent on auxiliary subunits and the Glu2A subtype [49,53].

AMPAR function is modulated by several auxiliary subunits such as the TARPs (transmembrane AMPAR regulatory proteins) [54]. Stargazin, the most representative TARP, is encoded by *CACNG2* and consists of four transmembrane domains and a cytoplasmic C-terminal with a PBM, essential for bridging the interaction between AMPAR and PSD-95 (Figure 1B) [54,55]. The association of the PSD-95 PDZ1 and -2 domains with the stargazin–AMPAR complex decreases the lateral mobility of AMPAR and increases its accumulation at the postsynaptic membrane, and thus, the amplitude of the excitatory postsynaptic potential (EPSC) [56,57,58].

Genetic variants in all four AMPAR subunits are linked to ID. Although *GRIA1* has primarily been associated with schizophrenia [59,60,61,62], a de novo variant identified in an individual with severe ID [63] was later found to be recurrent in six individuals with ID, ADHD, ASD, and/or delayed motor development [64]. *GRIA2* is implicated in ID [65,66,67] and ASD [68], and recently, 20 heterozygous de novo *GRIA2* variants were identified in 28 individuals with ID, ASD, and Rett syndrome-like features as well as seizures or DEE, suggesting that a defective GluA2 leads to NDD [69]. The AMPAR subunit gene most commonly associated with ID is *GRIA3*, which is also linked to ASD, global DD, seizures, and/or epileptic encephalopathy [51,70]. Functional analyses of a *GRIA3* missense variant leading to epileptic encephalopathy and global DD showed that mutant GluA3 receptors resulted in slower desensitization and deactivation kinetics in human embryonic kidney 293T (HEK293T) cells and slower ESPCs in neurons [71]. Through similar studies in HEK293 cells, four ID-associated *GRIA3* missense variants were shown to lead to protein misfolding, reduced channel function, or increased desensitization, and in all cases, altered kinetic properties [72]. A few reports have associated *GRIA4* variants with ID with or without seizures [65,73] and studies on *GRIA4* knockout mice suggest its involvement in the etiology of absence seizures [74,75]. Finally, variants in the auxiliary subunit gene *CACGN2* are implicated in ID [76] and in ASD [77] in single cases.

### 3.3. NMDA Receptors

Similar to AMPARs, NMDARs are ligand-gated cation channels found throughout the central nervous system. They play a role in nearly all brain functions and are critical for synaptic plasticity, development, and survival as well as learning and memory functions [78]. NMDARs are di- or triheterotetramers composed of two obligate GluN1 subunits (encoded by *GRIN1*), and either two GluN2 subunits (GluN2A-D, encoded by *GRIN2A-D*, respectively) or a mix of GluN2 and GluN3 subunits (GluN3A-B, encoded by *GRIN3A-B*, respectively; Table 1; Figure 1B) [79]. NMDAR subunits also contain four domains: NTD, LBD, TMD, and CTD, but are distinctive from other glutamate receptors in their high Ca^2+^-permeability, channel co-gating by glycine and glutamate (GluN1 and GluN3 LBDs bind glycine, GluN2 LBD binds glutamate), and voltage-dependent pore-blockage by extracellular Mg^2+^ [78,79]. These differences between NMDAR and AMPAR are crucial for their collective function in mediating EPSCs. Although they both bind glutamate, only AMPAR is activated upon glutamate release as Mg^2+^ blocks NMDAR. AMPAR-mediated depolarization displaces the Mg^2+^ blockage, allowing for NMDAR activation, which leads to a long-lasting synaptic current due to slower glutamate-unbinding, resulting in substantial Ca^2+^ influx [78,79]. This ultimately induces short- or long-term changes in synaptic strength, depending on the duration and frequency of NMDAR activation and subunit composition [78,80,81]. The latter, and particularly which GluN2 subtype (A-D) is incorporated into the NMDAR, is integral to its function, creating substantial NMDAR diversity and allows for fine-tuned regulation of synaptic strength and plasticity [78,79].

The CTDs of NMDAR subunits differ in length and are subject to alternative splicing and various PTMs [82]. They also contain a PBM (except in GluN3 subunits), which interacts with the PDZ1-2 domains of PDS-95 as well as a SH3 domain-binding motif, which associates with the SH3 domain of PSD-95, although this interaction is less studied [82,83]. This allows PSD-95 and other MAGUKs to anchor NMDARs in a subunit specific manner, essential for differential control of synaptic localization and stabilization of NMDARs (Figure 1B) [82].

All NMDAR subunit genes, except *GRIN3A-B*, are linked to NDDs and epilepsy. Early reports associated variants in *GRIN2A* and *GRIN2B* with ADHD [84,85], ID, and epilepsy [86]. Since then, more than 20 studies have linked variants in both genes to different NDDs and epileptic syndromes including ID, DD, ASD, movement disorders, seizures, epilepsies, and epileptic encephalopathy [87,88,89,90,91,92,93,94,95,96,97]. In addition, *GRIN2A* variants have been associated with aphasia, speech dysfunction, and Landau–Kleffner syndrome [98,99,100]. Although *GRIN2A* and *GRIN2B* are the NMDAR-genes most frequently associated with disease, variants in *GRIN2C* and *GRIN2D* have also been reported in individuals with ASD [90,101] and (developmental) epileptic encephalopathies [102,103], respectively. The obligate subunit GluN1 was first associated with disease in 2011 when de novo variants were identified in individuals with ID [76]. Additional studies have since supported the link to ID and furthermore associated *GRIN1* variants with early onset encephalopathy with or without epilepsy, DD, ASD, and polymicrogyria [96,97,104,105].

In the two extramembranous domains (NTD and CTD) of all NMDAR subunits, there is a higher number of presumably benign variants than in the LBD and TMD, where pathogenic variants are concentrated [97]. However, functional studies suggest that the variants affecting the ability of CTD to bind ligands are likely to be pathogenic. In one study, variants in the CTD of GluN2B reduced NMDAR surface expression due to impaired PSD-95 binding, although none of the investigated variants altered the PBM [93]. Similarly, protein-truncating variants in *GRIN2A* and *GRIN2B* decreased NMDAR surface expression and NMDAR-mediated currents in a haploinsufficient manner [89]. Although most protein-truncating variants result in nonsense-mediated decay (NMD), the authors speculated that a truncation of the CTD, which might escape NMD, could lead to similar phenotypes, partly due to reduced binding of CTD-ligands such as PSD-95 [89].

### 3.4. NLGN1

NLGNs are cell adhesion proteins on the postsynaptic membrane comprising NLGN1-4 and the less understood NLGN4Y, which is unable to properly localize to the membrane (Table 1) [106]. NLGN1 and NLGN4 are found in excitatory synapses, NLGN2 in inhibitory synapses, and NLGN3 in both [107,108]. They play a critical role in synapse maturation, spinogenesis, and brain function through their transsynaptic interaction with neurexins (Figure 1A) [107,109,110]. Each NLGN is composed of an extracellular acetylcholinesterase-like domain, a single transmembrane region, and a short cytoplasmic PBM-containing tail [111]. NLGN1 binds the third PDZ domain of PSD-95, which promotes NLGN1 clustering at excitatory synapses in a phosphorylation-dependent manner and transsynaptically modulates presynaptic neurotransmitter release probability [112,113,114].

Variants in genes encoding NLGNs, and in particular those in *NLGN3* and *NLGN4*, have repeatedly been associated with ASD [108]. Copy-number variants affecting *NLGN1* were found to be overrepresented in ASD individuals [115], and a partial deletion of the gene was detected in an individual with severe DD, seizures, and microcephaly [116]. Through exome sequencing, five *NLGN1* missense variants were identified in six ASD individuals, two of whom were siblings [117]. The variant identified in the siblings resulted in impaired spine formation and induced changes in subcellular localization and protein degradation. Recently, a homozygous nonsense *NLGN1* variant was detected in a monozygotic twin pair with ID and ASD [118]. In general, variants in *NLGNs* are considered highly penetrant, particularly for ASD phenotypes, which is speculated to be partially due to their PDS-95-dependent involvement in regulating the stability and trafficking of AMPAR and NMDAR [108].

### 3.5. ADAM22 and LGI1

The ADAM family members ADAM22, -23, and -11 lack the metalloprotease consensus sequence and are therefore catalytically inactive [119]. Instead, their relatively short cytoplasmic domain acts as a binding site for intracellular proteins, while the large extracellular part is involved in direct cell–cell signaling through ligand-binding [119]. When secreted into the synaptic cleft, the neuronal protein LGI1 acts as a ligand for ADAM22 (Table 1; Figure 1A) [120].

LGI1 and ADAM22 are major components of the PSD-95-complex as ADAM22 binds to the PDZ3 domain of PSD-95 through its C-terminal PBM [121]. In addition to LGIs and PSD-95, ADAM22 engages in complexes with ADAM23, ADAM11, K_v_1 channels, and other post- and presynaptic MAGUKs [6]. An expanded ADAM22 interactome includes NMDARs, AMPARs, and neurexins [6]. In fact, knockdown of ADAM22 or deletion of its PBM have shown a decrease in AMPAR- and NMDAR-mediated EPSCs in mice [122]. Here, AMPAR-mediated currents were dependent on the interaction of LGI1, ADAM22, and PSD-95, with the complex of the two former directing the role of the latter [122].

Autoantibodies against LGI1 are linked to seizures and cognitive dysfunction, and it is well established that variants in *LGI1* are causative for autosomal dominant lateral temporal lobe epilepsy [123,124,125]. Structural studies have shown that several of these variants affected the heterotetrameric assembly of the LGI1-ADAM22 complex [126]. Similarly, *ADAM22* variants identified in an individual with ID and seizures produced a protein unable to bind LGI1 [127] and a homozygous protein-truncating *ADAM22* variant detected in an individual with epilepsy and ID rendered ADAM22 incapable of associating with PSD-95 [6,128].

## 4. PSD-95 and Neurodevelopmental Disorders

Until our group recently described *DLG4*-related synaptopathy as a novel brain disorder, the association between dysfunctional DLGs and neurodevelopmental/neuropsychiatric conditions had been scarcely defined [11]. Few studies had linked *DLG4* variants to schizophrenia and NDDs such as ID and ASD [129,130,131,132]; *DLG2* variants to ASD, schizophrenia, and bipolar disorder [133,134,135]; and truncating *DLG3* variants to X-linked ID [136,137]. Identification of 45 different heterozygous *DLG4* variants in 53 individuals demonstrated that a deficient PSD-95 would lead to a brain disorder characterized by ID, global DD, ASD, ADHD, epilepsy, hypotonia, and movement disorders [11]. Brain magnetic resonance imaging (MRI) of the individuals with pathogenic *DLG4* variants revealed that the structural brain abnormalities were diverse and nonspecific. There is a general overlap between the clinical features of the genetic NDDs described in the previous section and *DLG4*-related synaptopathy. Therefore, as the alterations for PSD-95 or other PSD components are not disease specific, the most appropriate method for genetic diagnosis is clinical exome or whole genome sequencing and the evaluation of genes involved in ID and/or epilepsy (targeted ID- and epilepsy gene panels).

**Figure 2:**
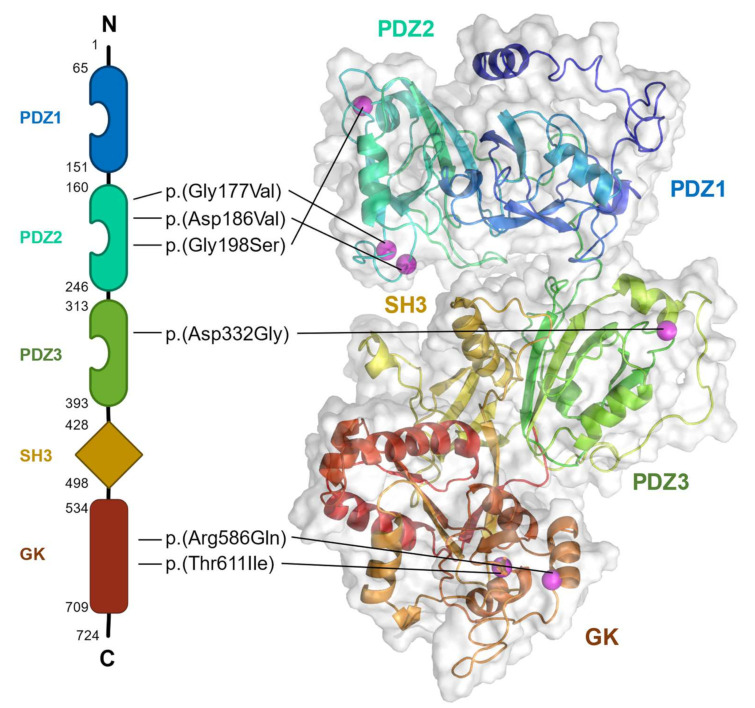
PSD-95 missense variants. Schematic (**left**) and structural (**right**) model of human PSD-95. The locations of the PDZ1, 2, and 3, SH3, and GK domains as well as the missense variants (p.(Gly177Val), p.(Asp186Val), p.(Gly198Ser), p.(Gly198Arg), p.(Asp332Gly), p.(Arg586Gln), and p.(Thr611Ile)) are indicated. Locations are described according to NP_001308004.1. N, N-terminal; C, C-terminal.

The majority of the *DLG4* variants are predicted to be protein-truncating and hereby LoF variants, indicating a cause–effect relationship between PSD-95 haploinsufficiency and the disease. Only six missense variants, all localizing to the highly conserved functional domains of PSD-95, have been reported. They are all predicted to be likely pathogenic, despite not directly altering the canonical binding pockets of the PDZ domains (Figure 2) [11]. Our group carried out molecular dynamics simulations, as such studies allow for reasonable approximation of the structural effects of missense variants in the absence of experimental data (Marcos-Alcalde et al., 2017). These analyses suggested that the *DLG4* missense variants affected either the surface electrostatic charge, the folding stability of the domains, or potential enzymatic activity [11]. Two of the missense variants, p.(Asp332Gly) and p.(Arg586Gln), residing in the PDZ3 and GK domains, respectively, were predicted to affect the electrostatic charge on the surface (Figure 2). p.(Asp332Gly) decreased the negative charge in a surface patch in the PDZ3 domain enriched with negatively charged residues, whereas p.(Arg586Gln) resulted in the emergence of a negatively charged patch in the GK domain (Figure 2). Both changes were suspected to affect PSD-95 interactions. p.(Gly177Val), p.(Asp186Val), and p.(Gly198Ser), all located in the PDZ2 domain, were predicted to affect the folding stability or the structure of the domain (Figure 2). p.(Gly198Ser) was predicted to disorganize an α-helix and modify an ubiquitylation recognition motif, while a likely benign variant affecting the same residue, p.(Gly198Arg), was predicted to not induce structural changes. p.(Thr611Ile), located in the GK domain, was predicted to affect the guanosine monophosphate (GMP) binding site, just as it would modify the structure of the putative active site (Figure 2).

The structure of PSD-95 is highly dynamic, and studies show that ligand-binding can induce inter- and intradomain rearrangements, which in turn can affect the ligands’ affinity to PSD-95 [13,14] suggesting a close link between PSD-95 structure and function. As the reported missense variants primarily affect the structure of the protein, it is likely that they also affect the interactions between PSD-95 and its numerous ligands, some of which are described above, and are essential for the PSD and thereby functional excitatory synapses.

## 5. The Pathogenesis of *DLG4* Missense Variants

Majority of the *DLG4* variants are predicted to lead to haploinsufficiency of PSD-95 resulting in a brain disorder, which suggests that the other members of the DLG subfamily are unable to compensate for PSD-95, as could have been expected. These LoF variants may result in a decreased function of several PSD-95 binding partners, some of which are described in the previous sections. It can, however, not be predicted through which mechanism (LoF or GoF) the *DLG4* missense variants exert their effect. We hypothesize that the symptoms presented in individuals with pathogenic *DLG4* variants may be caused by downstream effects of abnormal PSD-95 levels or function, particularly through the dysregulation of PSD-95 binding partners in the PSD.

PSD-95 anchors K_v_1 channels and the glutamate receptors NMDAR and AMPAR/stargazin to the postsynaptic membrane through their binding to the PDZ1 and PDZ2 domains (Figure 1). Three *DLG4* missense variants are located in the PDZ2 domain (Figure 2) and are predicted to affect the structure or folding ability of PSD-95, which may affect the binding of the interaction partners to the domain through disrupting the stability, and thereby the function. With regard to K_v_1 channels, genetic variants affecting the most abundant subunits were associated with NDD and epilepsy. Although it is unknown whether the function of K_v_1 channels is affected in individuals with *DLG4* variants, some of the symptoms may be due to decreased channel function. Similarly, variants in the genes encoding the subunits of both NMDAR and AMPAR as well as the important TARP, stargazin, were linked to epilepsies and NDDs. These glutamate receptors are essential for excitatory synaptic signaling and plasticity, and it is possible that loss (or maybe gain) of function of PSD-95 would affect receptor diffusion from the membrane, altering the postsynaptic nanodomains, and thereby causing a change in the amplitude of the EPSCs. This may result in symptoms similar to those linked to the variants identified in the glutamate receptor genes. In the case of NMDAR, it is plausible that potential variants in the SH3 domain of PSD-95, besides the variants in the PDZ1 and PDZ2 domains, could contribute to disease pathogenesis in a similar manner.

Both NLGN1 and the LGI1–ADAM22 complex are involved transsynaptic interactions (Figure 1A), and bind to PSD-95 domain PDZ3 where there is a single reported *DLG4* missense variant (Figure 2). This variant is predicted to decrease the negative charge in a negatively charged patch in the domain, likely affecting PSD-95 interactions. NLGN1 modulates presynaptic neurotransmitter release probability, and variants in *NLGN1* are associated with ASD and ID. Though speculative, it is possible that disruption of the interaction between PSD-95 and NLGN1 would affect synaptic transmission. The LGI1–ADAM22 complex, which has been linked to ID and seizures, is thought to modulate the role of PSD-95 in AMPAR- and NMDAR-mediated EPSCs. It is therefore unclear whether variants in the PDZ3 domain or loss of PSD-95 would affect the function of the complex.

Finally, there are two *DLG4* missense variants in the GK domain (Figure 2). These are predicted to affect either the surface electrostatic charge or enzymatic activity in the domain, and it is possible that these variants may exert their effect through dysfunctional binding to PSD-95 interaction partners more deeply embedded in the PSD. Disruption of the binding to SAPAP, which is part of the Shank–SAPAP–PSD-95 scaffolding complex connecting the cytoskeleton and the postsynaptic membrane (Figure 1A), would possibly affect the whole PSD architecture.

## 6. Final Remarks

There is a general overlap of the clinical symptoms associated with dysfunction of the different members of the PSD. Due to the complexity and extent of the PSD interactome, it is likely that the impairment of one member will affect several binding partners. Therefore, since PSD-95 is a central component in the PSD, crucial for orchestrating the architecture, it is possible that deficient PSD-95 can have a wide range of effects.

In addition to the described NDD, defective PSD-95 has also been associated with neurodegenerative disorders such as Alzheimer’s disease (AD). Here, the loss of PSD-95 from apical dendrites and accumulation in neuronal soma and Hirano bodies, which are protein aggregates associated with neurodegenerative disorders, have been suggested to inflict postsynaptic damage [138]. In a recent study, inhibition of depalmitoylation increased the synaptic levels of PSD-95, which protected the synapses from the toxic effect of amyloid-β peptides, a principal component in AD pathology [139]. This suggests that a pharmacological inhibition of depalmitoylation, increasing PSD-95 clustering at synapses, could be a potential therapeutic approach in AD treatment. As the majority of the individuals afflicted with *DLG4*-related synaptopathy are children or adolescents, it is currently unknown whether the pathogenic *DLG4* variants lead to neurodegeneration [11]. However, as most of the variants are predicted to be LoF, it may be hypothesized that decreased synaptic levels of PSD-95 result in postsynaptic damage and neurodegeneration later in life.

All in all, further studies are essential in order to elucidate how the pathogenic *DLG4* variants affect synaptic function. It would be of interest to utilize the missense variants as disease models and to investigate whether they induce structural changes and exert their effect indirectly through the PSD-95 binding partners above-mentioned. Furthermore, in the search for therapeutic strategies, it would be highly relevant to assess whether an increase in PSD-95 levels through pharmacological inhibition of depalmitoylation could alleviate some of the symptoms of the individuals with *DLG4* variants.

## Figures and Tables

**Table 1:** List of genes and proteins reviewed in this study.

Group	Gene	Protein	Alternative Names
DLG subfamily	*DLG1*	SAP97	
	*DLG2*	PSD-93	
	*DLG3*	SAP102	
	*DLG4*	PSD-95	SAP90
Kv1 channel subunits	*KCNA1*	Kv1.1	
	*KCNA2*	Kv1.2	
	*KCNA4*	Kv1.3	
NMDA receptor subunits	*GRIN1*	GluN1	NR1
	*GRIN2A*	GluN2A	NR2
	*GRIN2B*	GluN2B	
	*GRIN2C*	GluN2C	
	*GRIN2D*	GluN2D	
	*GRIN3A*	GluN3A	NR3
	*GRIN3B*	GluN3B	
AMPA receptor subunits	*GRIA1*	GluA1	GluR1
	*GRIA2*	GluA2	GluR2
	*GRIA3*	GluA3	GluR3
	*GRIA4*	GluA4	GluRA-D2
	*CACNG2*	Stargazin	TARP γ2
Neuroligins	*NLGN1*	NLGN1	
	*NLGN2*	NLGN2	
	*NLGN3*	NLGN3	
	*NLGN4*	NLGN4	NLGN4X
	*NLGN4Y*	NLGN4Y	NLGN5
LGI1 and ADAM22	*LGI1*	LGI1	Epitempin
	*ADAM22*	ADAM22	Mdc2

DLGs, discs large; K_v_1 channels, Shaker-type voltage-gated potassium channels; NMDA, *N*-methyl-D-aspartate; AMPA, α-amino-3-hydroxy-5-methyl-4-isoxazolepropionic acid; TARP, transmembrane AMPA receptor regulatory protein; LGI1, leucine-rich glioma-inactivated protein 1; ADAM22, a disintegrin and metalloprotease 22.

## Data Availability

Not applicable.
